# Rapid serotype-independent differential detection of biofilm-positive and biofilm-negative *Salmonella* using Fourier transform infrared biotyping

**DOI:** 10.1016/j.onehlt.2025.101004

**Published:** 2025-03-06

**Authors:** Asmita Shrestha, Smriti Shringi, Devendra H. Shah

**Affiliations:** Texas Tech School of Veterinary Medicine, 7671 Evans Drive, Amarillo, TX 79106, USA

**Keywords:** *Salmonella*, Biofilm, FTIR spectroscopy, IR Biotyper

## Abstract

Foodborne illnesses caused by *Salmonella* represent a global one health challenge, with biofilm-forming strains exhibiting enhanced public health risks due to their ability to persist due to resistance to antimicrobial agents, disinfectants, and environmental stresses. While food-safety and public health investigation primarily focus on *Salmonella* identification and source tracing, they often overlook the biofilm-forming capacity of isolates, limiting their predictive value for risks posed by biofilm producing *Salmonella*. This study assessed fourier transform infrared (FTIR) biotyping for rapid serotype-independent differentiatial detection of biofilm-positive (BFP) from biofilm-negative (BFN) *Salmonella*. A total of 270 *Salmonella* strains representing 12 common serotypes were classified using three conventional biofilm assays (congo red and coomassie brilliant blue agar test, calcofluor test, and tube test) into true BFP (*n* = 80), true BFN (*n* = 64), and uncertain (*n* = 59) biofilm producers. Biofilm production for each group was also assessed with a microtiter plate assay. FTIR biotyping was applied to a subset of 115 strains (61 BFP, 54 BFN). Using spectral windows of 1180–1050 cm^−1^ and 1400–1200 cm^−1^, FTIR biotyping accurately differentiated BFP from BFN strains with 93.4 % sensitivity, 83.3 % specificity, and 88.6 % overall accuracy. FTIR biotyping differentiated 59 strains with uncertain biofilm status into BFN (*n* = 45) and BFP (*n* = 14). FTIR biotyping provides a rapid, sensitive and specific method for detection of biofilm-forming *Salmonella* strains. Incorporating FTIR biotyping for biofilm detection in current *Salmonella* surveillance and source-tracing protocols can enhance food safety risk assessments and improve *Salmonella* outbreak prevention.

## Highlights

• *Salmonella*-induced foodborne illnesses are a global one health concern causing millions of infections and deaths annually.
• Biofilm-forming *Salmonella* pose greater public health risks due to enhanced resistance and persistence in the food chain.
• FTIR biotyping can rapidly and reliably differentiate biofilm-positive and biofilm-negative *Salmonella* strains.
• FTIR-based biofilm detection will improve risk assessment, and outbreak prevention via targeted *Salmonella* control.

## 1 Introduction

Foodborne illnesses can occur from the consumption of foods contaminated with a variety of adulterants including bacteria, parasites, viruses, toxins, or metals*.* Centers for Disease Control and Prevention (CDC) estimates that domestically acquired foodborne illnesses affect 48 million people annually in the United States, resulting in 128,000 hospitalizations, and 3000 deaths [1]*.* Among bacterial foodborne illnesses, non-typhoidal *Salmonella* causes an annual 1.35 million infections leading to 26,500 hospitalizations and 420 fatalities in the United States [1]. Globally, non-typhoidal *Salmonella* was estimated to cause between 31 and 211 million foodborne illnesses with 36,000 to 89,000 deaths in a single year [2]. The success of *Salmonella* as a foodborne pathogen underlies in its ability to efficiently traverse from the farm of origin to the consumer's kitchen via contaminated food by evading multiple food safety measures applied on farms, food processing plants, and kitchens, making this truly a one health challenge [3,4]. One of the most important factors that contribute to the persistence of *Salmonella* in the food system is its ability to form biofilms [5]. Biofilms produced by *Salmonella* enhance adherence and persistence on a wide range of biotic and abiotic surfaces by protecting this pathogen from antimicrobial agents, disinfectants, preservatives, and various other environmental stresses encountered throughout animal production, food processing, and storage [[6], [7], [8], [9], [10], [11]]. For instance, *Salmonella* hiding within biofilms can effectively survive treatment with disinfectants such as glutaraldehyde for up to 60 mins and ethanol for up to 10 mins, exhibit up to 200-fold greater resistance to clinically relevant antibiotics such as ciprofloxacin and can survive heat treatment at 80 °C for up to 20 mins [[12], [13], [14]].

Interestingly, however, not all *Salmonella* isolates produce biofilms. Few studies have revealed that 57 % - 92 % of *Salmonella* strains isolated from poultry and other food sources related to Salmonellosis outbreaks may produce varying degrees of biofilms with only 40 % - 54 % of isolates being detected as strong biofilm producers [[15], [16], [17], [18], [19]]. The improved survival and persistence of biofilm-forming *Salmonella* in animal production and processing environment significantly amplifies the risk of food contamination, thereby escalating the risk of foodborne illnesses. While the current *Salmonella* outbreak investigations primarily focus on *Salmonella* detection for source tracing [20,21] they often overlook the biofilm-forming capacity of isolates, limiting their predictive value for food safety and public health risks. Given the significant food safety and public health risks due to biofilm-associated *Salmonella,* rapid and reliable detection of biofilm-forming *Salmonella* can serve as a valuable food safety risk predictor, strengthen existing *Salmonella* surveillance, and more importantly, aid in the implementation of enhanced control measures for biofilm-producing *Salmonella*.

*Salmonella* biofilms are made of the extracellular polymeric substance (EPS) primarily consisting of a network of cellulose and curli [22]. Consequently, the conventional biofilm detection approaches in *Salmonella* have largely relied on phenotypic characterization and quantitative estimation of EPS from *Salmonella* incubated for 3 to 4 days on special media under biofilm-inducing conditions [23,24]. For instance, depending on the ability to produce cellulose and/or curli, *Salmonella* produces various colony morphotypes on congo red and coomassie brilliant blue (CRCBB) agar including red, dry, and rough (RDAR, both cellulose, and curli), brown, dry, and rough (BDAR, only curli), or pink, dry and rough (PDAR, only cellulose) after 4 days of incubation. In contrast, strains that don't produce either cellulose or curli yield a variety of phenotypes including smooth and white (SAW) or smooth, brown, and mucoid (SBAM). Similarly, the composition and quantity of EPS vary widely between *Salmonella* strains resulting in varying degrees of adherence to abiotic surfaces, which is often quantified by staining the amount of adhered *Salmonella* EPS in a conventional microtiter plate assay. These conventional approaches lack validation and suffer from several constraints such as time intensiveness, inconsistent and test-dependent variability inbiofilm detection rates, along with subjectivity of interpretation [25]. Therefore, there is a need to develop an objective, rapid, and reliable tool that can aid the differential detection of biofilm-forming *Salmonella* from non-biofilm former.

Fourier transform infrared (FTIR) spectroscopy is an analytical technique commonly used for structural elucidation and identification of microorganisms based on different functional groups within the complex biological matrix. When infrared (IR) radiation is bombarded onto biomolecules during FTIR spectroscopy, the biomolecules such as polysaccharides (cellulose), lipids, amino acids, and proteins absorb specific frequencies of the IR light, causing vibrations in their chemical bonds which correspond to bond stretching, bending, or twisting movements [26]. The FTIR spectroscopy measures the absorption of IR radiation at different wavelengths and generates a spectrum, that represents the unique vibrational fingerprints of the biomolecules allowing rapid and reliable discriminant analysis. As a result, this technique is now increasingly being evaluated for identification and typing bacterial strains such as *Klebsiella pneumoniae*, *Lactiplantibacillus plantarum,* and *Salmonella* [[27], [28], [29], [30]]. FTIR offers several advantages, including speed, reliability, objectivity, and cost-efficiency [31,32].

Recently, FTIR spectroscopy has been utilized in the detection of biomolecules such as curli fimbriae and cellulose [33,34]. Given that EPS of *Salmonella* is primarily composed of cellulose and curli, FTIR spectroscopy may offer a viable alternative for rapid and sensitive differential detection of biofilm-producing *Salmonella* from non-producing strains. Therefore, this study aimed to evaluate the potential of FTIR spectroscopy, specifically the IR biotyping, as a rapid method for distinguishing between biofilm-positive and biofilm-negative *Salmonella* strains. To achieve this, we tested the performance of FTIR spectroscopy using a comprehensive set of strains, including true biofilm-positive, true biofilm-negative, and strains with uncertain biofilm status, representing the twelve most prevalent *Salmonella* serotypes.

## 2 Materials and methods

### 2.1 Bacterial strains

Frozen glycerol stocks of 270 strains belonging to the 12 most prevalent *Salmonella* serotypes isolated from poultry were used in the study [35] These included *Salmonella* Enteritidis (*N* = 37), Hadar (*N* = 31), Heidelberg (*N* = 23), Infantis (*N* = 7), Kentucky (*N* = 55), Mbandaka (*N* = 26), Montevideo (*N* = 17), Schwarzengrund (N = 5), Senftenberg (*N* = 19), Thompson (*N* = 9), Typhimurium (N = 26), and 4,5,12,:i:- (*N* = 15).

### 2.2 Qualitative biofilm testing

#### 2.2.1 Congo Red Coomassie Brilliant Blue (CRCBB) agar test

CRCBB method is a standard laboratory technique used to assess biofilm production in *Salmonella* by growing on agar plates supplemented with congo red and coomassie brilliant blue dyes, where strong biofilm producers typically produce curli and /or cellulose and exhibit distinctively patterned aggregative colony morphotypes allowing researchers to evaluate the type of biofilm formation based on the appearance of the colonies on the plate. The colony morphotypes of 270 *Salmonella* strains associated with biofilm production were determined following the CRCBB test protocol described previously with minor modifications [9,36,37]. Briefly, frozen stocks of *Salmonella* were grown on MacConkey agar (cat #76461–576, VWR, USA) for 24  h at 37 °C. A single colony was suspended in 1 ml of Luria-Bertani broth without salt (LBWS) in a sterile 96-well block (cat #

22–038083, Bel-Art, USA) followed by incubation at 25 ± 2 °C for 16 h without shaking with a humidity range of 20–25 %. Using a micropipette, a 5 μl drop of overnight LBWS broth was dispensed in duplicate onto LBWS agar supplemented with Congo Red (40 μg/ml) (Sigma-Aldrich, USA) and Coomassie Brilliant Blue (20 μg/ml) (Sigma-Aldrich, USA), hereafter referred as CRCBB agar. The plates were incubated at 25 ± 2 °C for four days, and morphotypes were captured with the naked eye and light microscopy when they achieved a complete and stable phase [34,38]. Briefly, colonies on CRCBB agar were classified as smooth (shiny glistening surface) or rough (dull, bumpy, granular, or matte surface), and based on colour, colonies were classified as brown, red, white, or pink. Using a sterile toothpick, the colonies were classified as dry (brittle or powdery) or moist (not powdery) [39]. Based on the combination of these colony characteristics, the colony morphotypes were categorized as RDAR (red, dry, and rough), indicating the expression of both curli fimbriae and cellulose; PDAR (pink, dry, and rough), indicating the expression of cellulose but not curli; BDAR (brown, dry and rough), indicating expression of curli, but not cellulose, and SAW (smooth and white), indicating lack of expression of both cellulose and curli fimbriae, SBAM (smooth brown and moist), indicating possible capsule production without curli or cellulose, and SAP (smooth and pink) with an unknown composition [40,41].

#### 2.2.2 Calcofluor test

The calcofluor test is another standard method used to screen for biofilm production in *Salmonella* by growing on calcofluor stain-containing agar plates where calcofluor-binding of cellulose-producing *Salmonella* produces fluorescence under long-wave UV light, indicative of biofilm. Using a micropipette, overnight LBWS broth culture was dispensed in duplicate each as a 5 μl drop onto an LBWS plate supplemented with Calcofluor (20 mg/ml) (Sigma-Aldrich, USA) following protocol as described previously [42]. Plates were incubated at 25 ± 1 °C for 48 h and cellulose production was confirmed by observation of fluorescence upon brief exposure of colonies to the UV light (254/366 nm) using a hand-held UV lamp (Analytik Jena, US) in a dark room.

#### 2.2.3 Borosilicate tube test

The borosilicate tube test is also a commonly used method for the detection of biofilm-producing *Salmonella* where the occurrence of visible film at the liquid-air interface of the tube indicates biofilm formation. Strains from frozen stock were grown in a TSA plate overnight at 37 °C. A single colony was inoculated in 5 ml sterile LBWS broth in a borosilicate glass tube (cat #89090–312, Pyrex Vista, USA) and incubated at 25 ± 1 °C for 48 h. Each strain was tested twice. The air-liquid interface was grossly examined for the presence or absence of a slimy layer, indicating the status of biofilm production as positive or negative, respectively based on the previous protocol with few modifications [43].

### 2.3 Quantitative biofilm testing using microtiter plate test

Microtiter plate test assay is a quantitative colorimetric method commonly used to determine the biofilm biomass, with absorbance readings taken by a microplate reader. Biofilm quantitation of *Salmonella* strains was performed using a microtiter assay as described previously with minor modifications [44]. Strains were grown overnight at 37 °C in 96 well-blocks (Greinerbio-one, NC, USA), containing 1 ml of LB in each block. Bacterial cultures were transferred to 96-well flat-bottom polystyrene plates (cat #222–8030-01F, Evergreen Scientific, USA) containing sterile 200 μl LBWS (Difco, BD and company, Spark, MD, USA) via metal pronged replicator (Boekel Scientific, PA, USA). Reference strains *S.* Enteritidis G2 (negative control) and G3 (positive control) were included in each plate as assay controls. Four wells containing uninoculated LBWS were used as media control. After incubating plates at 25 ± 1 °C for 72 h without shaking, cultures of each well were decanted in 10 % bleach solution and wells were washed with 200 μl sterile 1× PBS three times to remove any loosely adhered bacteria. After drying wells at 37 °C for 20 mins, 250 μl of 0.1 % safranin (cat #477–73-6, Fisher Scientific) was added into each well and incubated for 15 mins at 25 ± 1 °C. Again, a single washing step was performed with 250 μl of 1× PBS. To dissolve the biofilm-bound safranin, 250 μl of 30 % acetic acid (cas # 64–19-7, Fisher Scientific, USA) was added to each well. Finally, OD_492_ nm was measured using an ELISA plate reader (Multiskan MCC, Fisher Scientific, USA). Each strain was tested independently at least three times. To normalize the data, the average OD_492_ value of media control was subtracted from all test strain OD_492_ values.

### 2.4 FTIR biotyping

For FTIR spectroscopy-based differential detection of biofilm-forming and non-forming *Salmonella*, a total of 61 true biofilm-positive (strain identified as positive based on CRCBB, calcofluor, and tube test) and 54 true biofilm-negative (strain identified as negative based on CRCBB, calcofluor, and tube test) strains were tested. Frozen glycerol stock was streaked onto the LBWS agar, incubated for 48 h at 28 °C, and processed according to the manufacturer's recommended protocol. Briefly, colonies directly from the confluent part of the culture were suspended into a 1.5 ml IRBT suspension *v*ial (IR Biotyper kit #1851760, Bruker, USA) containing 50 μl of 70 % (*v*/v) ethanol. The samples were homogenized by vortexing for 30–60 s. The homogenous suspension was diluted with 50 μl of sterile miliQ water followed by vortexing for 30 s. A 15 μl of this homogeneous suspension was spotted uniformly in triplicates on a silicon IR Biotyper plate. Samples on the spots were allocated according to the manufacturer's template for 25–30 samples. For controls, 90 μl of sterile deionized water and absolute ethanol were added to IRTS 1 and IRTS 2 standards and vortexed. Finally, 12 μl of IRTS 1 and IRTS 2 were spotted onto their pre-designated spots on the IR Biotyper plate covering the entire spots evenly, without leaking out. The plate was incubated at 37 °C for 10–30 min for drying and then scanned using IR Biotyper. Spots for which spectra passed validation status were utilized for further evaluation. Each strain was tested in three technical replicates to generate 360 spectra from a total of 115 strains. Wavenumber threshold percentages from 15 % to 50 % were used to empirically select the optimal range of wavenumbers for splicing as described in the manufacturer's instruction manual (IR Biotyper Software User Manual Revision F). The dendrograms showing hierarchical clustering analysis using an unweighted pair group method with an arithmetic mean (UPGMA) model, were generated using the OPUS software *V* 8.2 and the IR Biotyper Client Software V3.0 (Bruker, Germany).

Data Analysis:

Data from the qualitative and quantitative tests was compiled, organized, and analyzed using MS-EXCEL and R software (4.3.1). Based on the results of the CRCBB, calcofluor, and tube test, the strains were broadly categorized into three groups: true biofilm-positive, true biofilm-negative, and uncertain strains. The difference in the median OD_492_ values between the three biofilm groups was analyzed by the Kruskal-Wallis test. The performance of FTIR biotyping for the differential detection of biofilm-positive and biofilm-negative strains was evaluated using multiple statistical metrics. Sensitivity and specificity were used to quantify the true positive and true negative detection rates, while positive predictive value (PPV) and negative predictive value (NPV) assessed the test's predictive power. The odds ratio was computed to estimate the strength of the association between FTIR biotyping results and biofilm status. Youden's index, a comprehensive measure of test performance, was also derived to summarize the discriminatory power of the technique. The performance of FTIR biotyping for the differential detection of strains with uncertain biofilm status into biofilm-positive or biofilm-negative was evaluated by comparing a training set of true biofilm-positive and true biofilm-negative strains with a representative challenge set of strains with uncertain biofilm status using IR Biotyper Client Software V3.0 (Bruker, Germany). The likelihood of each CRCBB morphotype being associated with either the biofilm-positive (BFP) or biofilm-negative (BFN) FTIR cluster was calculated as follows: likelihood of BFP cluster: P(BFP) = (Number of strains in BFP) / (Total number of strains (N)) and likelihood of BFN cluster: P(BFN) = (Number of strains in BFN) / (Total number of strains (N)) [45].

## 3 Results and discussion

### 3.1 Biofilm categorization of *Salmonella* strains based on conventional qualitative tests

**Fig. 1:**
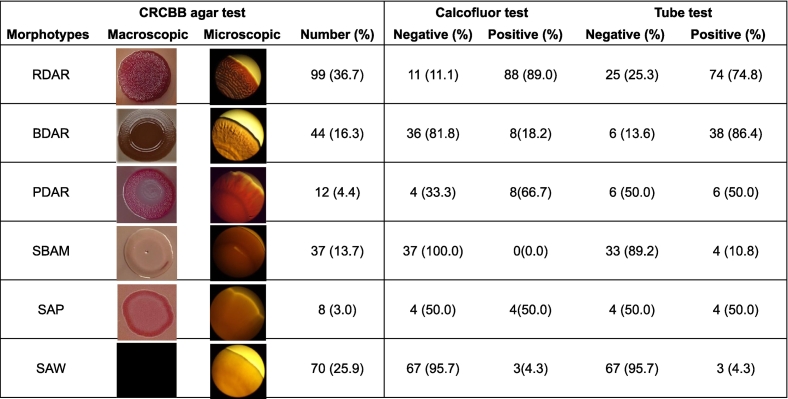
Distribution of biofilm morphotypes among 270 *Salmonella* strains representing 12 serotypes based on three conventional assays. Representative images showing six distinct CRCBB colony morphotypes after 96 h of incubation at 25 ± 1 °C. Microscopic images were captured at 4× magnification under a light microscope. The calcofluor and tube test results for strains within each of the CRCBB morphotypes are included for comparison.

A total of 270 *Salmonella* strains were categorized into six biofilm phenotypes based on the colony morphotypes observed on the CRCBB agar (Fig. 1). These morphotypes included red, dry, and rough [RDAR; 36.7 % (99/270)], brown dry and rough [BDAR; 16.3 % (44/270)], pink, dry and rough [PDAR; 4.4 % (12/270)], smooth, brown and moist [SBAM; 13.7 % (37/270)], smooth and pink [SAP; 3 % (8/270)], and smooth and white [SAW; 25.9 % (70/270)]. RDAR morphotype has been previously reported to produce strong biofilms made of a network of both cellulose and curli whereas the SAW colony morphotype does not produce either cellulose or curli and thus, is considered biofilm non-producer [4,42]. BDAR and PDAR morphotypes have been reported as moderate or weak biofilm producers with variable production of only curli or only cellulose, respectively [36,40,46,47]. In contrast, SBAM and SAP are not commonly reported colony morphotypes, however, these have been reported in some studies as either weak or non-biofilm-producing strains due to a lack of both cellulose and curli [14,48,49]. Collectively, according to the CRCBB test, a total of 155 (54.4 %) strains including 99 (36.7 %) RDAR, 44 (16.3 %) BDAR, and 12 (4.4 %) PDAR morphotypes could be classified as strong to moderate biofilm producers. On the other hand, 37 (13.7 %) SBAM and 8 (3 %) SAP strains could be classified as weak biofilm producers whereas 70 (25.9 %) SAW strains could be classified as biofilm non-producers. In contrast, according to the calcofluor test, 111 (41.1 %) strains produced fluorescence due to cellulose production and thus were identified as biofilm-producers whereas 159 (58.9 %) strains failed to produce fluorescence likely due to lack of cellulose production leading to the identification of these strains as biofilm non-producers (Fig. 1). Finally, in the tube test, 129 (47.8 %) strains produced pellicles at the liquid-air interface and thus were identified as biofilm-producers whereas 141 (52.2 %) strains were identified as biofilm non-producers due to lack of pellicle formation. As expected, the results revealed wide variation in the outcomes of individual qualitative tests in their ability to discriminate strains as biofilm producers or non-producers (Supplementary file 1). For instance, 88.9 % of RDAR morphotypes were identified as biofilm producers by calcofluor test, however, only 74.7 % of these strains were identified as biofilm producers by tube test. Similarly, 81.8 % of BDAR morphotypes were identified as biofilm non-producers by calcofluor test, but 86.4 % as biofilm producers by tube test (Fig. 1).

**Fig. 2:**
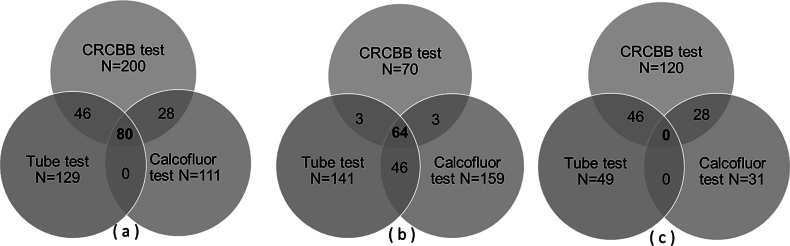
Distribution of true biofilm-positive (a), true biofilm-negative (b), and uncertain strains (c) among 270 *Salmonella* strains representing 12 serotypes. N indicates total number of strains positive by specific tests in Fig. 2(a) and (c), whereas negative by specific tests in Fig. 2(b). True biofilm-positive (BFP) strains (*n* = 80) were identified as consistently producing biofilms in all three biofilm assays. In contrast, true biofilm-negative (BFN) strains (*n* = 64) consistently tested negative in all three tests. The strains (*n* = 126) with inconsistent results were identified as strains with uncertain (U) biofilm status (c).

Given the wide variation in the results of conventional phenotypic assays, we combined the results of all three phenotypic assays to identify sets of true biofilm-positive (BFP) and true biofilm-negative (BFN) strains for downstream testing by FTIR biotyping. This yielded a set of 80 true BFP strains that were consistently identified as biofilm-producers by CRCBB, calcofluor, and tube test (Fig. 2a), and a set of 64 true BFN strains that were consistently identified as biofilm non-producers by CRCBB, calcofluor, and tube (Fig. 2b). In contrast, 126 strains that did not meet either of these two criteria were classified as strains with uncertain (U) biofilm status (Fig. 2c).

### 3.2 Quantitative assessment of biofilm production by *Salmonella* strains

**Fig. 3:**
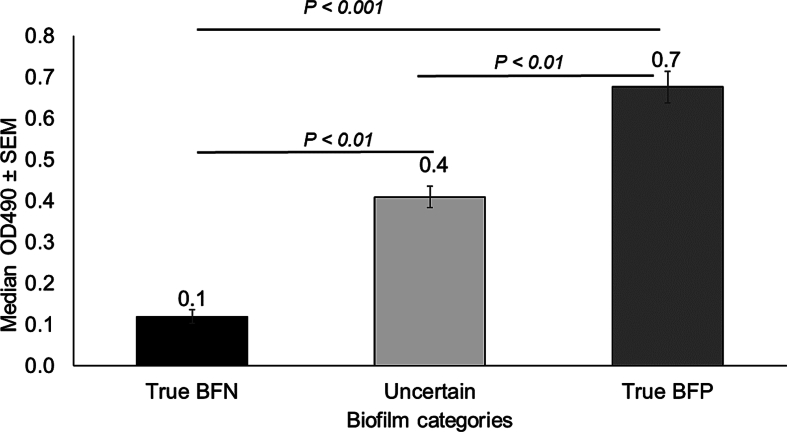
The median OD_490_ values for true biofilm-negative (n = 64), uncertain (n = 126), and true biofilm-positive (n = 80) groups of *Salmonella* strains. Error bars represent the standard error of the mean (SEM). The differences in median OD_490_ among the three biofilm class groups (true BFN, true BFP, and Uncertains) were statistically significant (*P* < 0.01, Kruskal-Wallis test, and pairwise rank sum test with Bonferroni correction).

To further confirm biofilm production by strains identified within true BFP (*n* = 80), true BFN (*n* = 64), and U (*n* = 126) biofilm groups, we determined the adherence of these strains to polypropylene surface using a quantitative microtiter plate test (Fig. 3). The results of this test revealed that the median OD_490_ for true BFP strains (0.68 ± 0.35) was significantly higher when compared with true BFN strains (0.12 ± 0.13). In contrast, the median OD_490_ for strains with U biofilm status (0.41 ± 0.29) was significantly lower than the true BFP strains but higher than true BFN strains (Fig. 3). A Kruskal-Wallis rank sum test revealed statistically significant differences in median OD values between the groups, χ^2^ (2) = 109.6, *p* < 2.2e-16. Furthermore, post-hoc pairwise rank-sum tests conducted with Bonferroni correction revealed significant differences between BFP and BFN (*p* < 2.e-16), BFP and uncertain (*p* = 8.5e-09), and BFN and uncertain (*p* = 1.4e-11). These data show that the strains identified as true BFP indeed produce strong biofilms whereas strains identified as true BFN do not produce biofilm. In contrast, strains identified as U biofilm status likely have a mixed population of weak biofilm producers and biofilm non-producers.

### 3.3 Differential detection of true BFP and true BFN *Salmonella* by FTIR biotyping

**Fig. 4:**
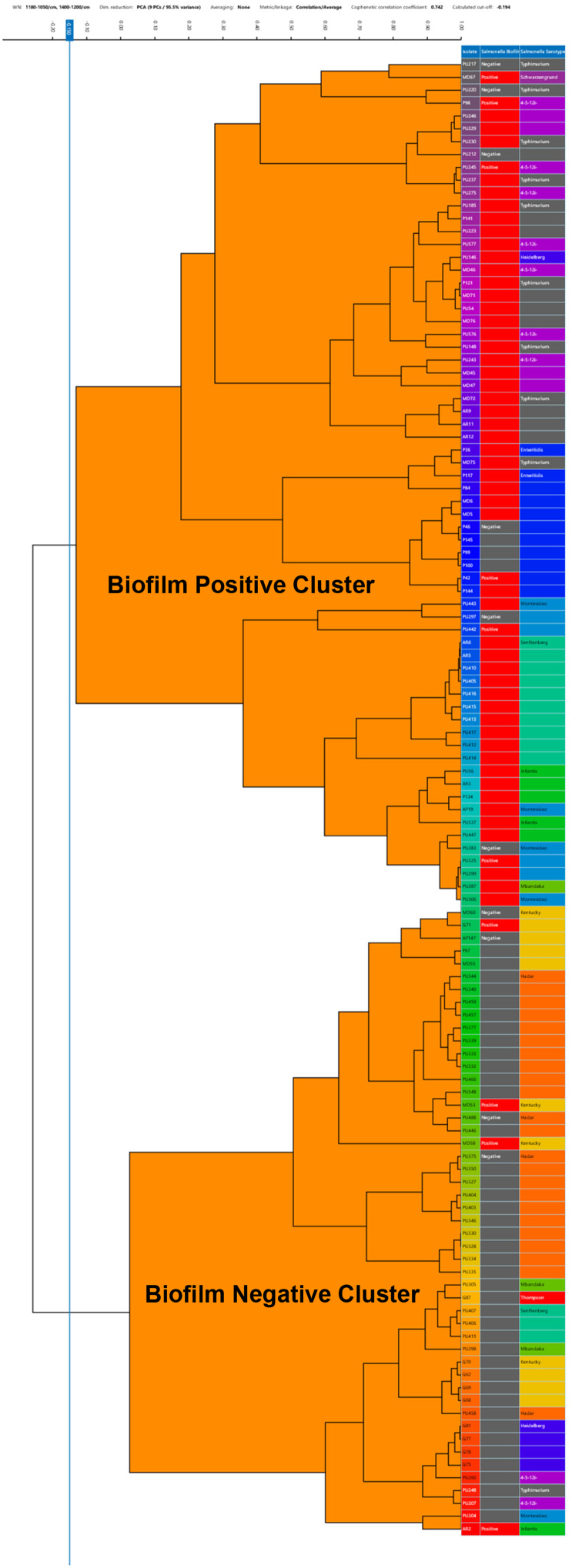
Dendrogram displaying the hierarchical clustering analysis results for 345 spectra of 115 true BFN (*n* = 54) and true BFP (*n* = 61) *Salmonella* strains representing 12 different serotypes from three measurements, using the wavenumber-window of 1180–1050 and 1400–1200 cm^−1^ and correlation and unweighted pair group method with arithmetic mean (UPGMA) for clustering. The automatically calculated cutoff value is −0.154 and is indicated as a vertical blue line. The columns at the end represent the strain ID (first column), biofilm production status (second column with grey representing BFN and red representing BFP), and serotype of the strain (different colors represent different serotypes). (For interpretation of the references to colour in this figure legend, the reader is referred to the web version of this article.)

To determine if FTIR biotyping can reliably detect and differentiate between true BFP and true BFN strains in a serotype-independent manner, we conducted FTIR biotyping of a set of 61 true BFP and 54 true BFN strains representing all 12 serotypes of *Salmonella*. To achieve optimal discriminatory power, we evaluated several FTIR spectral wavenumbers (WN) windows with a threshold range from 15 % to 50 % (Table 1). The spectral WN windows 1180–1050 cm^−1^, 1180–1010 cm^−1^, 1310–1280 cm^−1^, 1450–1200 cm^−1^, and 1500–955 cm^−1^ yielded the most discriminatory dendrograms displaying hierarchical clustering analysis using correlation and unweighted pair group method with arithmetic mean (UPGMA)*.* The sensitivity, specificity, and Youden index for distinct clusters generated using each spectral WN window were calculated (Table 1). The number of false positives and false negatives varied for each combination of spectral WN windows used. The dendrogram created using the spectral WN-windows 1180–1050 and 1400–1200 cm^−1^ optimally clustered true BFP and true BFN strains in two distinct clusters with sensitivity and specificity of 0.93, and 0.83, respectively (Fig. 4). The BFP cluster included a majority of true BFP strains (57/61, 93.4 %) suggesting that the FTIR biotyping correctly classifies 93.4 % of the true BFP strains as BFP with a false negative rate of 6.6 %. Four true BFP stains that clustered within the BFN cluster included one strain of *S. infantis* (RDAR) and three strains of *S.* Kentucky with one strain exhibiting BDAR and two strains exhibiting RDAR colony morphotype. Similarly, the BFN cluster included a majority of true BFN strains (45/54, 83.3 %) suggesting that FTIR biotyping correctly identified 83.3 % of the non-biofilm-producing *Salmonella* strains with a false positive rate of 16.7 % (Table 1). Nine true BFN strains that clustered within the BFP cluster included *S.* Enteritidis (*n* = 4), *S.* Montevideo (*n* = 2), and *S.* Typhimurium (*n* = 3), with all strains exhibiting SAW morphotype on the CRCBB test. The optimal diagnostic performance of the FTIR biotyping was determined based on the highest Youden's index for the selected spectral WN-windows 1180–1050 and 1400–1200 cm^−1^ using the ROC package in R 4.3.1 (Table 1). The results revealed that the bands captured using spectral WN windows 1180–1050 and 1400–1200 cm^−1^ provide fairly reliable performance in classifying *Salmonella* strains as BFP or BFN in a serotype-independent manner with an overall accuracy of 88.6 % (95 % CI range 81 % - 93 %). In general, most of the bands that occur in the IR spectra of microorganisms can be assigned to vibrational modes that correlate with specific chemical structures. Spectral wavenumber window 1300–1000 cm^−1^ is dominated by characteristic absorptions from functional groups including C—O, C—C, and C-O-C, and plane bends of C—H bonds possibly in ethers, alcohols, and carbohydrates [50,51]. Among these, 1042.44 cm^−1^ is particularly dominated by absorptions from C—O stretch in polysaccharides such as cellulose extracted from the biofilm of *Salmonella* [34,52], and 1150–950 cm^−1^ from sugar compounds [51]. Similarly, absorption bands from spectra of bacterial cellulose between the range of 1180–1050 cm^−1^ were particularly associated with the C1-O-C4 functional group linked to the glycosidic bond of cellulose, and C2-O2H and C3-O3H pyranose ring vibrations typical of cellulose [53,54]. Finally, spectral WN window 1400–1200 cm^−1^ is dominated by absorptions from mixed regions. Based on the wavenumbers that yielded the most discriminatory power, it appears that polysaccharides such as cellulose, curli, and other mixed molecules produced by *Salmonella* likely served as underlying functional groups for the FTIR spectroscopy-based differential detection of true BFP and true BFN.

**Table 1:** Sensitivity, specificity, and Youden's index for different spectral wavenumber windows used for differential detection of true BFP and true BFN *Salmonella* strains by FTIR biotyping.

Wavenumbers (cm^−1^)	Splicing Methods				
	1	2	3	4	5
	1450–1200	1180–1010	1180–1050	1310–1280	1180–1010
	1180–1050	1500–955	1400–1200	1180–1050	1500–955
	–	–	–	–	1310–1280
False Positive	9	8	9	9	9
False Negative	4	6	4	5	5
Sensitivity	0.93	0.90	0.93	0.92	0.92
Specificity	0.83	0.85	0.83	0.83	0.83
Youden's Index	0.77	0.75	0.77	0.75	0.75
Positive Predictive Value	0.86	0.87	0.86	0.86	0.86
Negative Predictive Value	0.92	0.88	0.92	0.9	0.9
Positive Likelihood Ratio	5.61	6.08	5.61	5.51	5.51
Negative Likelihood Ratio	0.08	0.11	0.08	0.1	0.1
Odds Ratio	71.25	52.71	71.25	56	56

### 3.4 Evaluation of FTIR biotyping for differential detection of *Salmonella* strains with uncertain biofilm status

**Fig. 5:**
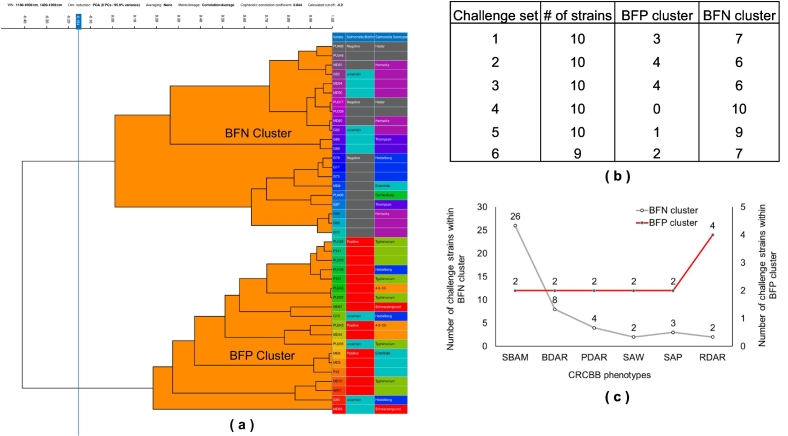
Dendrogram showing hierarchical clustering analysis of challenge set 1 consisting of ten *Salmonella* strains with uncertain biofilm status along with a training set of 15 *Salmonella* strains each representing true biofilm-positive and true biofilm-negative groups using correlation and UPGMA. The columns at the right of the cluster represent the strain ID (first column), biofilm production status (second column with grey representing BFN, red representing BFP, and sky blue representing uncertain status), and serotype of the strain (third column with different colors representing different serotypes). Using a consistent cut-off of 0.15, four strains with U biofilm status clustered within the BFP and six strains with U biofilm status clustered within the BFN cluster. Using a similar approach, five additional challengesets of 9 or 10 strains each with U biofilm status were tested (see supplementary file 2). The cluster distribution of these additional strains from each challenge set (b) and the distribution of CRCBB morphotypes among the challenge strain sets (c) are shown for comparison. (For interpretation of the references to colour in this figure legend, the reader is referred to the web version of this article.)

We evaluated the efficiency of FTIR biotyping to classify *Salmonella* strains with U biofilm status into BFN or BFP using 59 *Salmonella* strains with U biofilm status. To accomplish this objective, we created a random set of 9 to 10 strains with U biofilm status as a challenge set and conducted FTIR biotyping in comparison with a subset of 15 true BFP and 15 true BFN as a standard training set. The hierarchical clustering analysis of challenge strain sets with U biofilm status revealed that 45/59 (76.3 %) *Salmonella* strains clustered within the BFN cluster whereas 14/59 (23.7 %) clustered within the BFP cluster. A total of 28 (47.5 %) out of 59 strains with U biofilm status exhibited an SBAM morphotype that lacks cellulose and curli. The majority of these strains (26/28, 92.9 %) clustered with the BFN cluster. The SBAM morphotype has been occasionally reported in *S.* Kentucky and a few strains of *S.* Typhimurium, *S.* Enteritidis, *S*. Montevideo, *S.* Mbandaka, *S.* Infantis, and a few other rare serotypes [[55], [56], [57], [58]]. It has been suggested that these strains may loosely adhere to abiotic surfaces due to capsular polysaccharides but fail to form mature biofilms [55,59]. The quantitative microtiter assay results also revealed that the OD_490 (0.24)_ of *Salmonella* strains exhibiting SBAM colony morphotypes was higher than OD_490 (0.1)_ of true BFN, but significantly lower than the OD_490 (0.7)_ of true BFP strains (Fig. 3). Therefore, the strains that exhibited SBAM morphotype either do not form biofilm or may only adhere loosely to form immature biofilms that are highly fragile and easily dispersible [42,59]. These results may explain almost unambiguous clustering within the BFN cluster and suggest that FTIR biotyping is correctly identifying these strains as true BFN. Ten (17 %) out of 59 strains with U biofilm status exhibited BDAR morphotype with two (20 %) of these strains clustered within the BFP cluster while eight (80 %) clustered within the BFN cluster. Strains with the BDAR morphotype are known to produce curli fimbriae, but either don't produce or exhibit weak cellulose production. Consequently, strains with BDAR morphotype are often reported as weak or moderate biofilm producers [42,60]. Therefore, the FTIR clustering of strains with BDAR morphotype within the BFN cluster is not surprising. Collectively, these data show that FTIR biotyping consistently classified the majority of strains with SBAM and BDAR morphotypes with U biofilm status as BFN. The remainder of the 23 strains with U biofilms status clustered within either BFN or BFP cluster varied in their CRCBB morphotypes including PDAR (*n* = 6), RDAR (n = 6), SAW (*n* = 4), SAP (*n* = 5), and SBAM (*n* = 2) (Fig. 5).

### 3.5 Likelihood of CRCBB morphotype being classified by FTIR biotyping in BFP or BFN cluster

The likelihood analysis was conducted to evaluate the effectiveness of FTIR biotyping in the differential detection of *Salmonella* strains classified into BFP and BFN categories. The FTIR biotyping demonstrated a robust capability to accurately cluster these strains based on their potential to form biofilm. A total of 91.9 % of strains with RDAR morphotype were classified as BFP by FTIR biotyping, underscoring the significant roles of both curli and cellulose in facilitating biofilm formation in this morphotype which likely aided accurate detection by FTIR biotyping (Table 2). Conversely, 75 % of strains with the BDAR morphotype were classified as BFN, highlighting that despite the presence of curli, the lack of cellulose in these strains likely allowed accurate identification of these strains as BFN. For the PDAR morphotype, 55.6 % of strains were classified as BFP, and 44.4 % were classified as BFN. All PDAR strains classified as BFP tested positive on the calcofluor test suggesting these strains produced cellulose. In contrast, 3 out of 4 strains with PDAR morphotypes that clustered with BFN tested negative on the calcofluor test, indicating that differences in cellulose production in these strains may have resulted in accurate differential detection of these strains by FTIR biotyping. Additionally, 92.9 % of the strains with SBAM morphotype were unambiguously classified as BFN, which can be attributed to the lack of both cellulose and curli. Similarly, the majority of strains (81 %) with the SAW morphotype were predominantly classified as BFN, reinforcing the impact of curli and cellulose absence on the accurate classification of these strains as BFN. The strains with SAP morphotype are not expected to produce cellulose or curli, however, these strains variably clustered with BFN and BFP, with 40 % of strains classified as BFP. Collectively, there was good concordance between CRCBB phenotype and the ability of FTIR biotyping to classify strains as either BFP or BFN.

**Table 2:** Likelihood differential detection of CRCBB morphotypes of *Salmonella* strains into biofilm-positive and biofilm-negative by FTIR biotyping.

CRCBB morphotypes	N	Curli	Cellulose	BFP cluster	BFN cluster	Likelihood (BFP)	Likelihood (BFN)
RDAR	62	Present	Present	57	5	0.92	0.08
BDAR	12	Present	Absent	3	9	0.25	0.75
PDAR	9	Absent	Present	5	4	0.56	0.44
SBAM	28	Absent	Absent	2	26	0.07	0.93
SAP	5	Absent	Absent	2	3	0.40	0.60
SAW	58	Absent	Absent	11	47	0.19	0.81

In conclusion, the results highlight the potential of FTIR biotyping for the rapid, differential detection of biofilm-positive and biofilm-negative *Salmonella* with high sensitivity, specificity, and accuracy. This method offers a more efficient and reliable tool for detecting biofilm-producing *Salmonella* compared to conventional phenotypic techniques. The timely detection of biofilm-forming *Salmonella* strains using FTIR is particularly important from a one health perspective, as biofilm formation can enhance the survival and persistence of *Salmonella* across diverse environments, from agricultural settings to food production and human health. Biofilm-producing strains pose significant challenges not only to food safety but also to public health, as they are more resistant to cleaning protocols, and antimicrobial treatments, and can persist in both animal and human reservoirs. Early detection of biofilm status using FTIR biotyping can aid in design and implementation of targeted and effective control measures, preventing the transmission of these resilient pathogens across the human-animal-environment interface. By facilitating more informed and rapid responses, FTIR biotyping has the potential to mitigate the risks associated with biofilm-producing *Salmonella* and support mitigation efforts for this global one health challenge.

## Ethics statement

No ethical approval was required for this research work.

## Funding

This study was funded by the Texas Tech University School of Veterinary Medicine.

## CRediT authorship contribution statement

**Asmita Shrestha:** Methodology, Investigation, Formal analysis, Writing – original draft. **Smriti Shringi:** Supervision, Resources, Methodology, Conceptualization, Writing – review & editing. **Devendra H. Shah:** Supervision, Resources, Project administration, Investigation, Funding acquisition, Formal analysis, Conceptualization, Writing – review & editing.

## Declaration of competing interest

The authors declare no competing interests.

## Data Availability

No data was used for the research described in the article.

## Appendix A Supplementary data

Supplementary material 1 Image 1

Supplementary material 2 Image 2
