# Supplementary material for: Rapid serotype-independent differential detection of biofilm-positive and biofilm-negative Salmonella using Fourier transform infrared biotyping
Source: One Health. 2025 Mar 6;20:101004. doi: 10.1016/j.onehlt.2025.101004 (PMC11931386; doi:10.1016/j.onehlt.2025.101004)
Supplement: Supplementary file 2 — Supplementary material 2 [file mmc2.pdf]

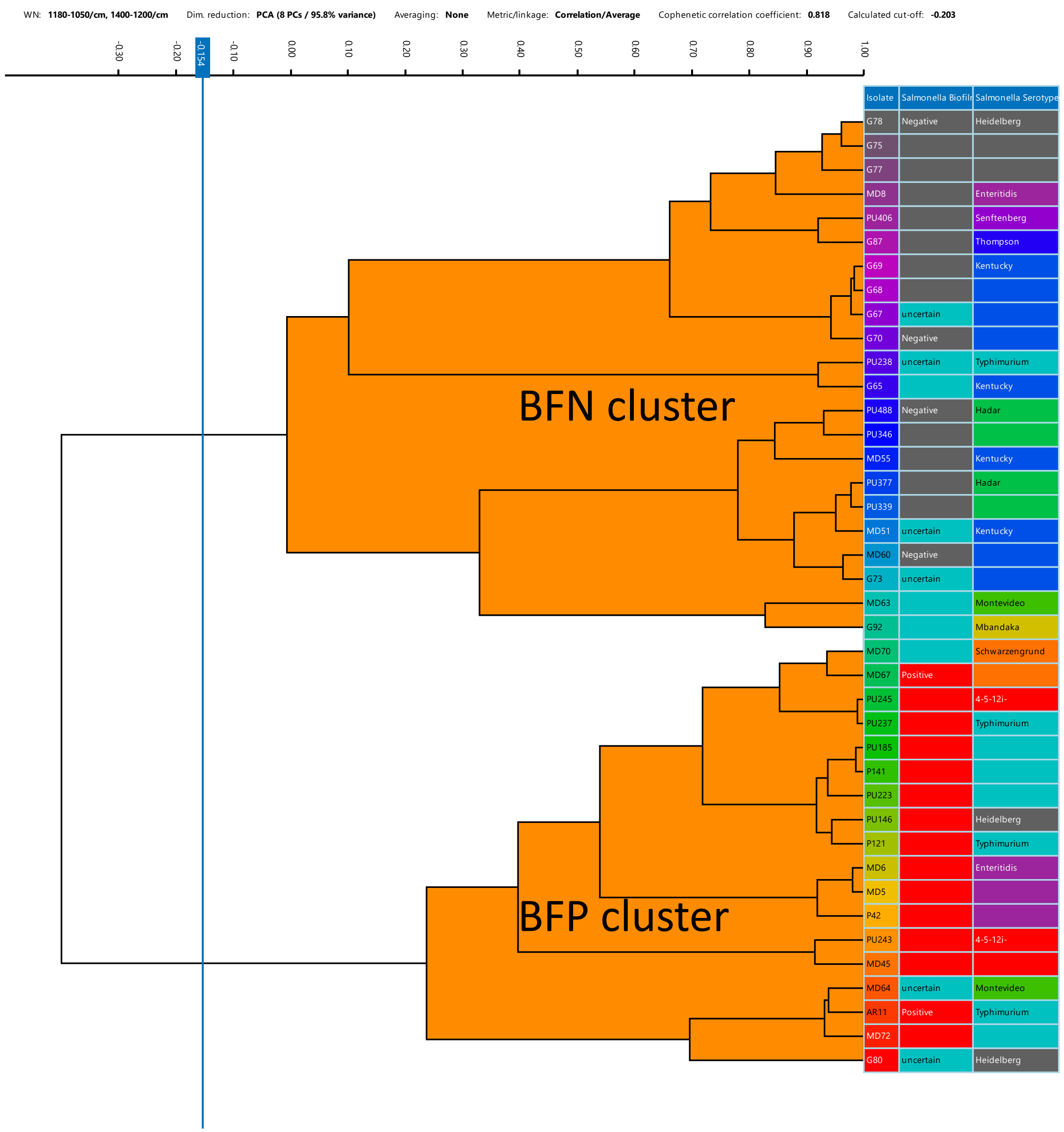

Supplementary Fig S1. Dendrogram showing hierarchical clustering analysis of challenge set 2 consisting of ten *Salmonella* strains with uncertain biofilm status along with a training set of 15 *Salmonella* strains each representing true biofilm-positive and true biofilm-negative groups using correlation and UPGMA. The columns at the right of cluster represent, the strain ID (first column), biofilm production status (second column with grey representing BFN, red representing BFP and sky blue representing uncertain status), and serotype of the strain (third column with different colors represent different serotypes). Using a consistent cut-off of 0.15, two strains with U biofilm status clustered within the BFP and eight strains with U biofilm status clustered within the BFN cluster.

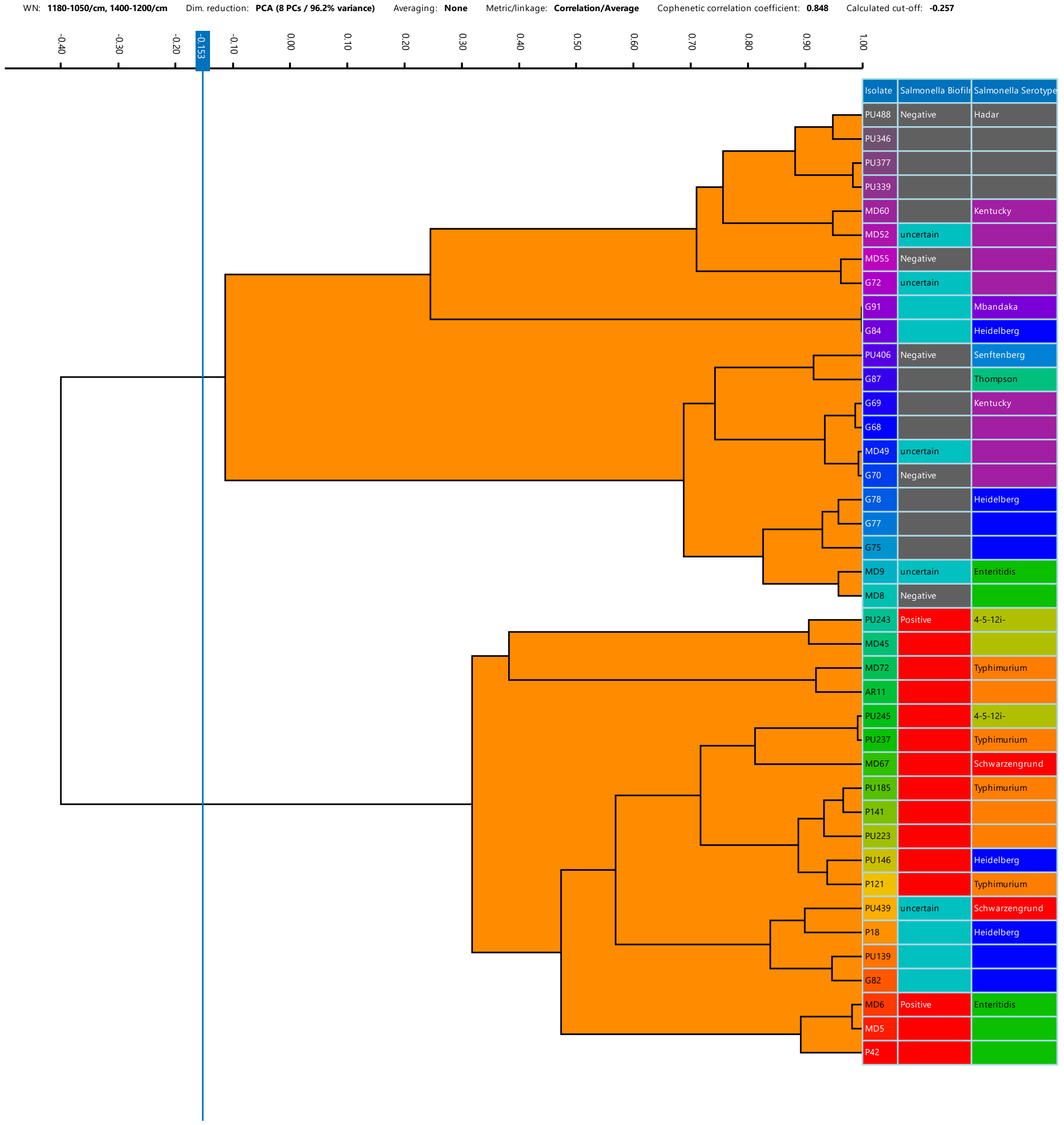

Supplementary Fig S2. Dendrogram showing hierarchical clustering analysis of challenge set 3 consisting of ten *Salmonella* strains with uncertain biofilm status along with a training set of 15 *Salmonella* strains each representing true biofilm-positive and true biofilm-negative groups using correlation and UPGMA. The columns at the right of cluster represent, the strain ID (first column), biofilm production status (second column with grey representing BFN, red representing BFP and sky blue representing uncertain status), and serotype of the strain (third column with different colors represent different serotypes). Using a consistent cut-off of 0.15, four strains with U biofilm status clustered within the BFP and six strains with U biofilm status clustered within the BFN cluster.

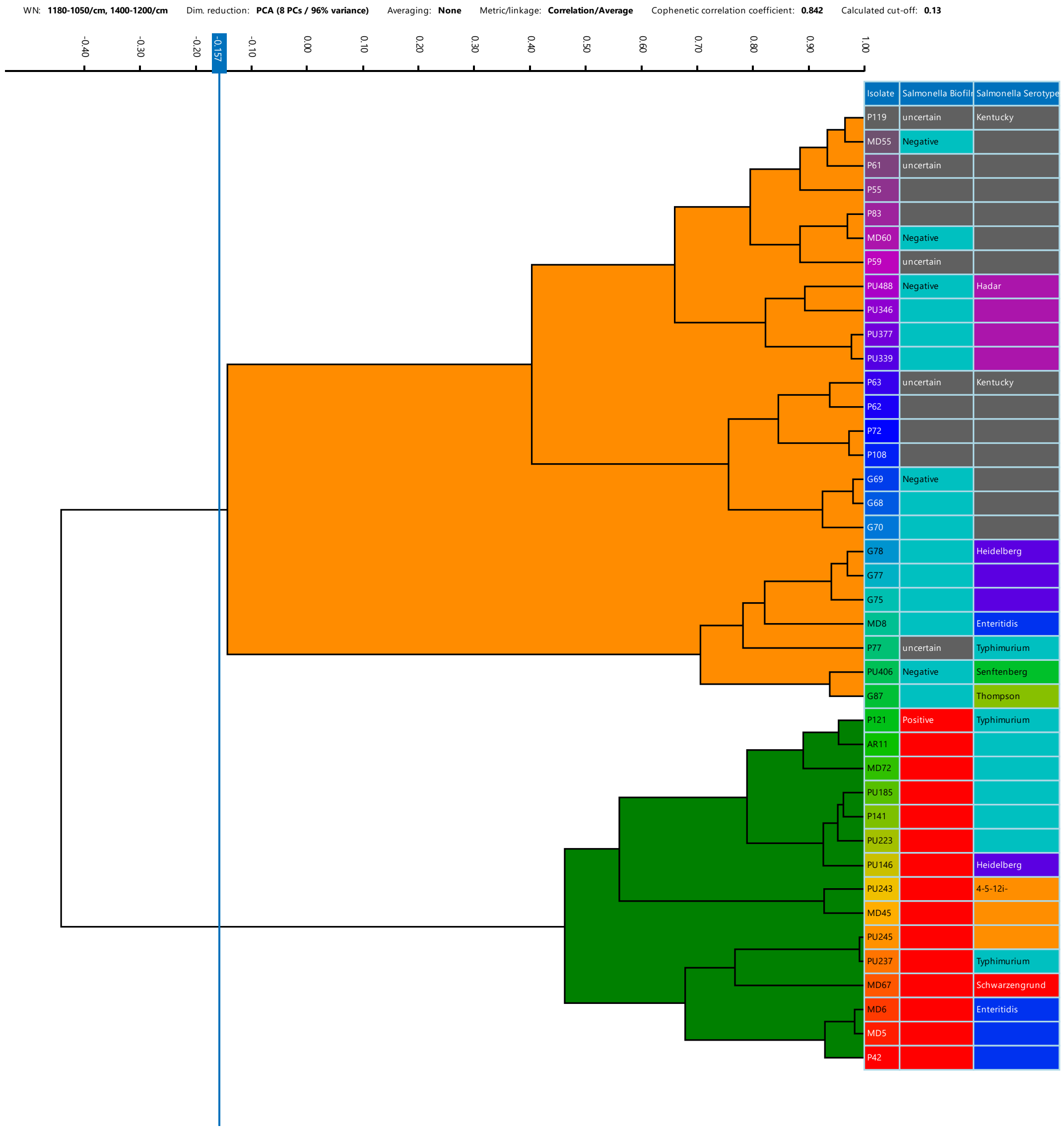

Supplementary Fig S3. Dendrogram showing hierarchical clustering analysis of challenge set 2 consisting of ten *Salmonella* strains with uncertain biofilm status along with a training set of 15 *Salmonella* strains each representing true biofilm-positive and true biofilm-negative groups using correlation and UPGMA. The columns at the right of cluster represent, the strain ID (first column), biofilm production status (second column with sky blue representing BFN, red representing BFP and grey representing uncertain status), and serotype of the strain (third column with different colors represent different serotypes). Using a consistent cut-off of 0.15, none of the strains with U biofilm status clustered within the BFP and all ten strains with U biofilm status clustered within the BFN cluster.

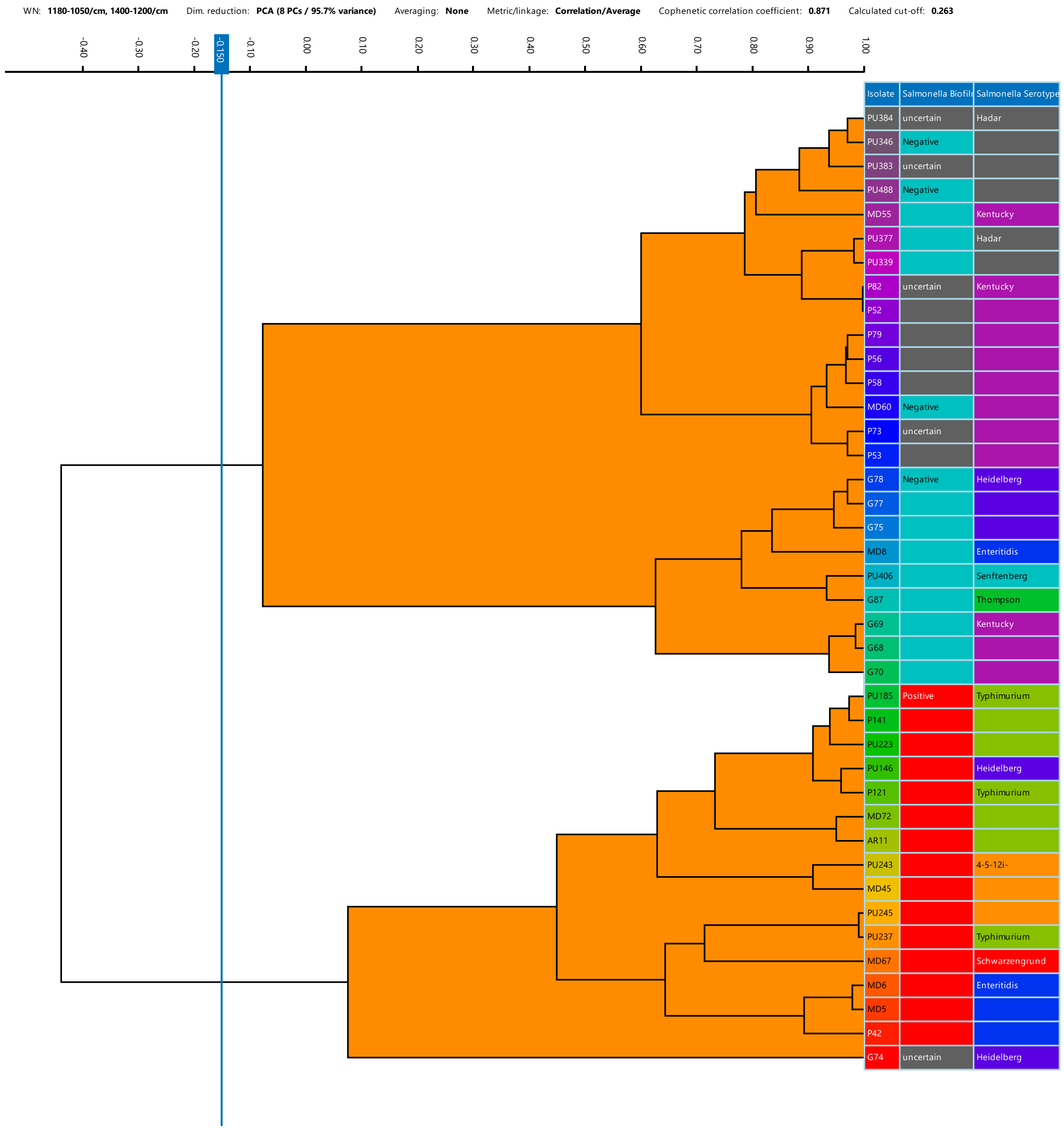

Supplementary Fig S4. Dendrogram showing hierarchical clustering analysis of challenge set 2 consisting of ten *Salmonella* strains with uncertain biofilm status along with a training set of 15 *Salmonella* strains each representing true biofilm-positive and true biofilm-negative groups using correlation and UPGMA. The columns at the right of cluster represent, the strain ID (first column), biofilm production status (second column with sky blue representing BFN, red representing BFP and grey representing uncertain status), and serotype of the strain (third column with different colors represent different serotypes). Using a consistent cut-off of 0.15, one of the strains with U biofilm status clustered within the BFP and nine strains with U biofilm status clustered within the BFN cluster.

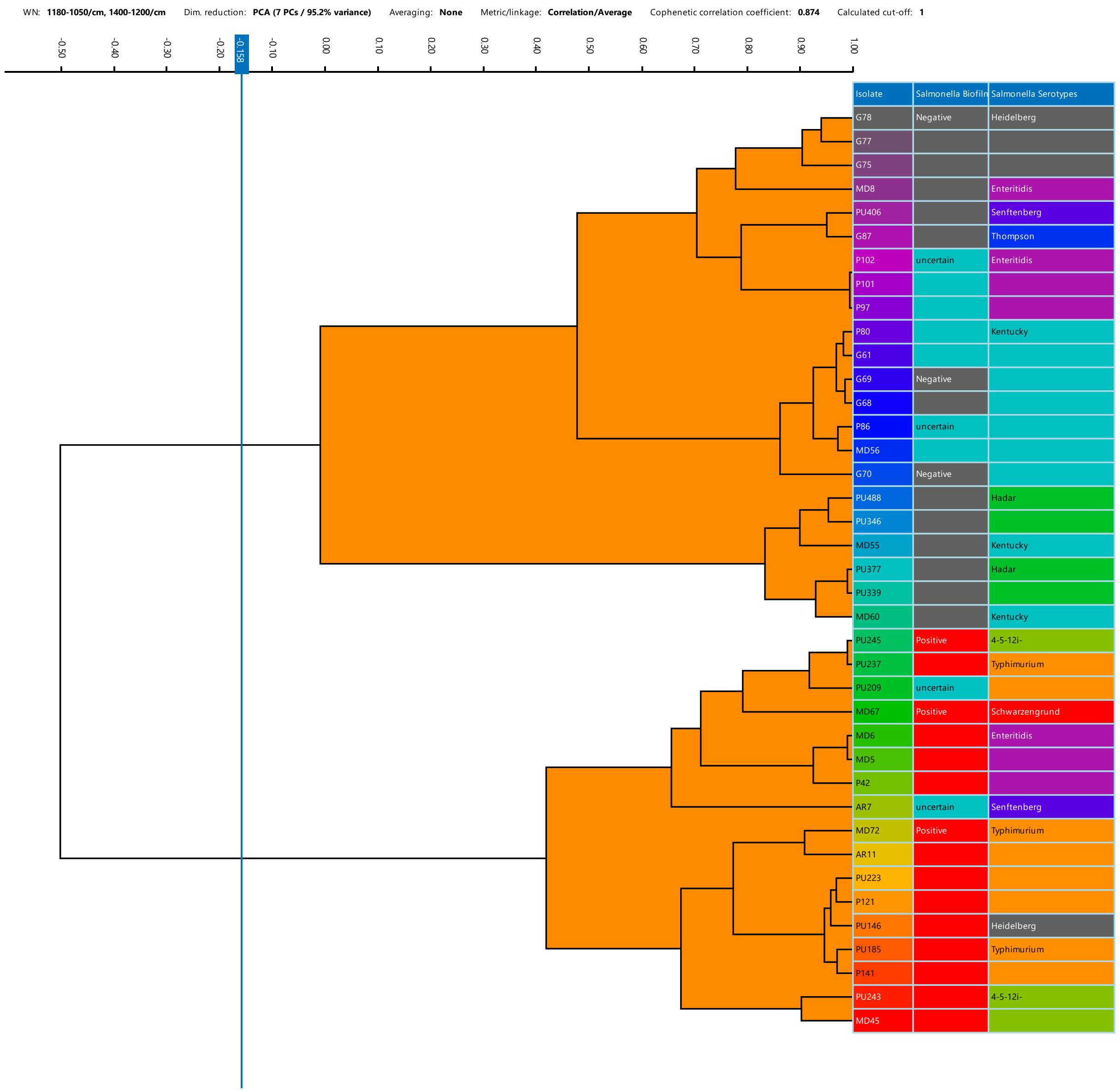

Supplementary Fig S5. Dendrogram showing hierarchical clustering analysis of challenge set 2 consisting of nine *Salmonella* strains with uncertain biofilm status along with a training set of 15 *Salmonella* strains each representing true biofilm-positive and true biofilm-negative groups using correlation and UPGMA. The columns at the right of cluster represent, the strain ID (first column), biofilm production status (second column with sky blue representing BFN, red representing BFP and grey representing uncertain status), and serotype of the strain (third column with different colors represent different serotypes). Using a consistent cut-off of 0.15, two of the strains with U biofilm status clustered within the BFP and seven strains with U biofilm status clustered within the BFN cluster.
